# Probing the Validity of the Zintl−Klemm Concept for Alkaline-Metal Copper Tellurides by Means of Quantum-Chemical Techniques

**DOI:** 10.3390/ma13092178

**Published:** 2020-05-09

**Authors:** Sabrina Smid, Simon Steinberg

**Affiliations:** Institute of Inorganic Chemistry, RWTH Aachen University, Landoltweg 1, D-52074 Aachen, Germany; sabrina.smid@rwth-aachen.de

**Keywords:** tellurides, polar intermetallics, bonding analyses

## Abstract

Understanding the nature of bonding in solid-state materials is of great interest for their designs, because the bonding nature influences the structural preferences and chemical as well as physical properties of solids. In the cases of tellurides, the distributions of valence-electrons are typically described by applying the Zintl−Klemm concept. Yet, do these Zintl−Klemm treatments provide adequate pictures that help us understanding the bonding nature in tellurides? To answer this question, we followed up with quantum-chemical examinations on the electronic structures and the bonding nature of three alkaline-metal copper tellurides, i.e., NaCu_3_Te_2_, K_2_Cu_2_Te_5_, and K_2_Cu_5_Te_5_. In doing so, we accordingly probed the validity of the Zintl−Klemm concept for these ternary tellurides, based on analyses of the respective projected crystal orbital Hamilton populations (−pCOHP) and Mulliken as well as Löwdin charges. Since all of the inspected tellurides are expected to comprise Cu−Cu interactions, we also paid particular attention to the possible presence of closed-shell interactions.

## 1. Introduction

Tellurides, which are compounds [1,2] that are considered to comprise of at least one tellurium atom in a reduced state relative to its elemental form, have attracted an enormous interest among chemists, physicists, and engineers. Namely, the impetus to explore the chemical and physical properties of tellurides is stimulated by the circumstance that several tellurides are at the cutting edge of many fields of basic research including those efforts focusing on thermoelectrics [3,4], phase-change data storage materials [5,6], and topological insulators [7]. Yet, there is still a critical need to even accelerate the discoveries of materials showing the desired chemical and physical properties in light of the growing demand [8] for sustainable materials addressing future challenges. In this connection, computational materials design [9,10] that is based on combining high-throughput density-functional-theory-based approaches with intelligent data mining has emerged as beneficial means.

Understanding the information that is derived from the density-functional-theory-based computations also requires a proper knowledge of the nature of bonding in solid-state materials. Namely, the bond energy has a significant contribution to the total (electronic ground state) energy [11,12], and the bonding characteristics at the Fermi level of a given material provide valuable hints accounting for its properties. The former aspect is extremely relevant for the evaluations of the total energies in order to justify and predict the formations of solid-state materials [13,14], while the bonding nature at the Fermi level, for instance, accounts for the presence of superconducting states in certain chalcogenides [15,16], magnetic ground states in transition-metals [17], or occurrence of vacancies in phase-change materials [18]. In the cases of the tellurides, the valence-electron distributions are typically determined by applying the Zintl−Klemm concept, which has originally [19,20] been introduced to understand correlations between structural features and valence-electron distributions in intermetallics composed of main group elements. Since its introduction, the Zintl−Klemm formalism that predicts an electron transfer from a more electropositive to a more electronegative element has been applied to numerous intermetallics, which were not only composed of main group elements but also contained transition-metals (TM)—for some cases in light of hypervalency [21,22]. In the spirit of this formalism, the interactions between tellurium atoms and early as well as late TM atoms are frequently described [23,24,25,26] as ionic and covalent, respectively. Yet, more recent research [27,28,29,30] on the electronic structures of certain tellurides, particularly, their nature of bonding, showed that such pictures of the bonding nature should be regarded with concern. Hence, do such Zintl−Klemm treatments help us understanding the nature of bonding in these particular cases, and how else should the bonding nature in such tellurides be described?

**Figure 1:**
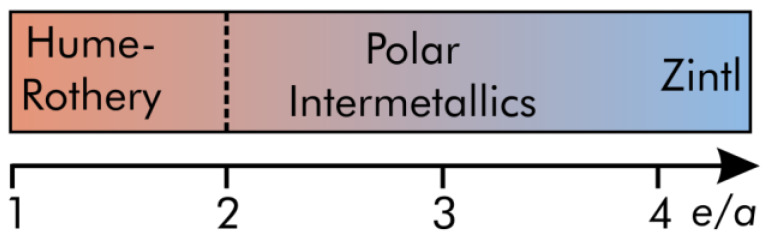
Overview [12] of different families of intermetallic phases as function of the valence-electron concentration (*e*/*a*).

The aforementioned quantum-chemical examinations [27,28,29,30] of the electronic structures of these tellurides containing (early and) late transition-metals revealed that the TM−tellurium states are well populated such that the majorities of the bonding interactions reside between these states. Since this outcome is indicative of an absence of full valence-electron transfers (like in ionic bonds) from the TM to the tellurium atoms, it was concluded that the TM−tellurium interactions should be depicted as strong (polar) mixed-metal-like bonds rather than ionic bonds. In view of the outcome of these quantum-chemical explorations and the aforementioned reports on ionic early-TM−Te interactions, it was concluded that the valence-electron distributions as suggested by the Zintl−Klemm treatments could be misleading and, hence, should be regarded with concern. Additional research [29,30] on the electronic structures of tellurides comprising both alkaline-metal−tellurium and transition-metal−tellurium contacts showed that the alkaline-metal−tellurium states indeed show characteristics of rather ionic bonds. Additionally, the same research identified the presence of late TM−late TM interactions, which could not be predicted by the Zintl−Klemm treatments. Due to the aforementioned bonding situation, it was inferred that these tellurides exhibit attributes of polar intermetallics [31,32] rather than Zintl phases that are typically related to higher valence-electron concentrations (*e*/*a*) than the polar intermetallics (Figure 1).

The outcome of these bonding analyses on tellurides containing lanthanides also raises the general question if tellurides comprising transition-metals should be assigned to the group of the Zintl phases, which are traditionally composed of main group elements, or polar intermetallics. Hence, where is the frontier between the group of polar intermetallics and the family of the Zintl phases? To answer this question, we followed up with a bonding analysis for NaCu_3_Te_2_ [33], K_2_Cu_2_Te_5_ [34], and K_2_Cu_5_Te_5_ [35], which do not comprise any lanthanides in contrast to our previous explorations [29,30] and short Cu−Cu contacts. As we will show below, applying the Zintl−Klemm formalism to these ternary tellurides will predict electron-precise distributions of valence-electrons for them; however, to reveal the electron transfers from the metal to the tellurium atoms, and, hence, the bonding nature between these contacts, quantum-chemical efforts are needed. Therefore, we examined the densities-of-states, projected crystal orbital Hamilton populations, and Mulliken as well as Löwdin charges for these tellurides, which are presented as an outcome of our explorations in the present contribution.

## 2. Computational Details

Chemical bonding and Löwdin as well as Mulliken population analyses were accomplished to determine the nature of bonding and distributions of valence-electrons in NaCu_3_Te_2_, K_2_Cu_2_Te_5_, and K_2_Cu_5_Te_5_. Prior to the bonding and population analyses, full structural optimizations, which included lattice parameters and atomic positions, were carried out utilizing the projector augmented wave (PAW) [36] method as implemented in the Vienna ab-initio simulation package (VASP) [37,38,39,40,41]. In addition to the structural optimizations, the aforementioned density-functional-theory-based method was also used to compute electronic band structures. In all computations, correlation and exchange were described by the generalized gradient approximation of Perdew, Burke, and Ernzerhof (GGA−PBE) [42] and the energy cutoff was set to 500 eV. Sets of 18 × 18 × 3, 4 × 8 × 4, and 12 × 3 × 3 *k*-points were used to sample the first Brillouin zones in NaCu_3_Te_2_, K_2_Cu_2_Te_5_, and K_2_Cu_5_Te_5_, respectively. All computations were considered to be converged, as the energy differences between two iterative steps of the electronic (and ionic) relaxations fell below 10^−8^ (and 10^−6^) eV/cell.

The bonding analyses were accomplished based on the projected crystal orbital Hamilton populations (−pCOHP) [43] and their respective integrated values (−IpCOHP). In the −pCOHP approach that is a variant of the −COHP [44,45] technique, the off-site densities-of-states (DOS) are weighted with the respective Hamilton matrix elements to identify antibonding, non-bonding, and bonding interactions. To determine the distributions of the valence-electrons in the tellurides, the Mulliken and Löwdin charges [46] were computed by subtracting the gross population of a given sort of atom from its number of valence-electrons. Since the computations of the –pCOHP and the Mulliken as well as Löwdin charges require the uses of local basis sets, the delocalized nature of the electronic structures within the plane-waves had to be transferred into a local one with the aid of transfer matrices. These transformations, which resulted in the constructions of the crystal wave functions from the plane-wave-based computations, were accomplished with employing the local orbital basis suite towards electronic-structure reconstruction (LOBSTER) [43,44,47,48] code. The DOS and –pCOHP curves of NaCu_3_Te_2_, K_2_Cu_2_Te_5_, and K_2_Cu_5_Te_5_ (shown below) were plotted with the aid of the wxDragon [49] program. In addition, the cumulative –IpCOHP/cell values that are the sums of all –IpCOHP/bond values for one particular type of interaction within a given unit cell were projected as percentages to the net bonding capabilities to reveal similarities but also differences between the electronic structures of the different tellurides. A direct comparison between the –IpCOHP values of dissimilar compounds cannot be made, because the electrostatic potential of each density-functional-theory-based computation scales to an arbitrary “zero energy”, whose relative position may vary from system to system—a circumstance that has also been described in detail elsewhere [45].

## 3. Results and Discussion

To explore the bonding nature of Cu−Cu interactions in tellurides, which are expected to comprise such closed-shell interactions, we carried out bonding analyses for the ternary NaCu_3_Te_2_, K_2_Cu_2_Te_5_, and K_2_Cu_5_Te_5_, whose crystal structures have been determined by previous experimental research [33,34,35]. Before presenting the results of our quantum-chemical explorations, we will provide brief overviews regarding the structural features of these tellurides.

**Figure 2:**
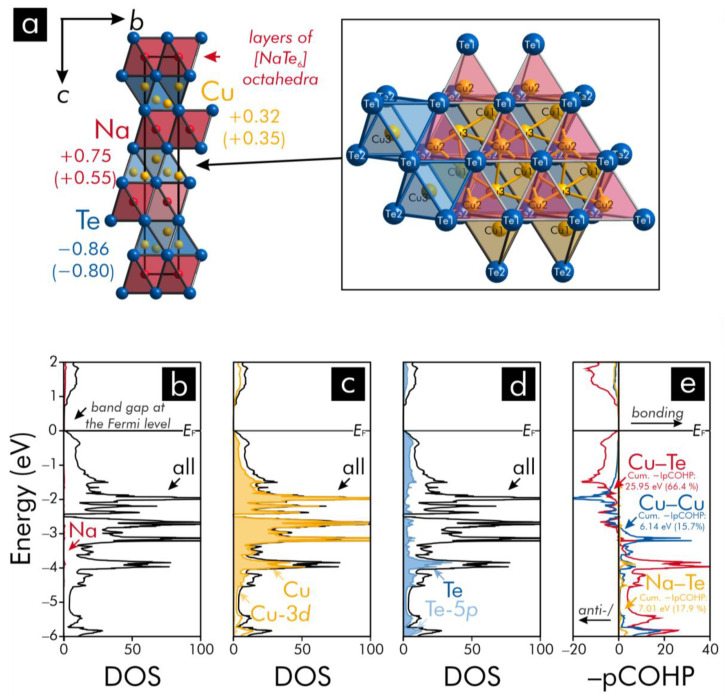
(**a**) Representation of the crystal structure of NaCu_3_Te_2_: the diverse types of tellurium polyhedra surrounding the copper atoms are shown in the inset, while the respective averaged Mulliken and Löwdin charges (in parentheses) have been included; (**b**–**d**): atom- and orbital-projected densities-of-states (DOS) curves: the horizontal lines indicate the highest occupied states below a band gap between the valence band maximum and the conduction band minimum. The orbital-projected DOS shown in the Figure 2 correspond to those states with the largest contributions to the DOS near the Fermi level, *E*_F_; (**e**): projected crystal orbital Hamilton population (pCOHP) curves: the cumulative –IpCOHP/cell values and their percentages to the net bonding capabilities have been included.

The crystal structure of NaCu_3_Te_2_, which has recently been subject to explorations on topological semi-metals [50,51], (Figure 2) comprises three independent copper positions: the copper atoms corresponding to two of these sites (Cu1 and Cu2) reside in the centers of tellurium tetrahedra, while the Cu3 atoms occupy octahedral voids enclosed by the tellurium atoms; however, the Cu3 atoms are evidently displaced from the centers of these octahedral voids such that each Cu3 atom rather assembles a trigonal pyramid with three tellurium atoms of one of the faces of each octahedron. Since the indices of the tellurium atoms in every copper-containing unit (denoted by the square brackets in our contribution) refer to the respective numbers of tellurium atoms surrounding the copper atoms, the tellurium units containing the Cu3 atoms will be represented by the formula [Cu@Te_6_] in the following. A detailed inspection of the coordination environments for the Cu1 and Cu2 atoms reveals that each [Cu1@Te_4_] tetrahedron and each [Cu2@Te_4_] tetrahedron are linked via common tellurium vertices to nearest neighboring [Cu1@Te_4_] and [Cu2@Te_4_] units, respectively. These nets of the [Cu1@Te_4_] and [Cu2@Te_4_] tetrahedra are condensed via common edges to layers with the Cu3 atoms occupying the octahedral voids in the aforementioned layers. As an outcome, the [Cu@Te_4_] tetrahedra and [Cu@Te_6_] units are condensed via common faces to layers that are stacked along the *c* axis and comprise remarkably short Cu−Cu separations (<3.09 Å) between the copper atoms of the diverse units. Under consideration of the covalent radius [52] of Cu (1.32 Å), such Cu−Cu distances point to the possible presence of Cu−Cu interactions, which will be probed by the following bonding analysis. The sodium atoms are encapsulated by tellurium octahedra, which are condensed to sheets between the layers of the [Cu@Te_4_] and [Cu@Te_6_] units. An application of the Zintl−Klemm concept to this ternary telluride results in an electron-precise valence-electron distribution according to the formula (Na^+^)(Cu^+^)_3_(Te^2−^)_2_; however, the Mulliken and Löwdin charges (Figure 2) of copper and tellurium differ evidently from those charges predicted by applying the Zintl−Klemm concept.

An examination of the DOS and –pCOHP curves (Figure 2) for NaCu_3_Te_2_ confirms the outcome derived from the Mulliken and Löwdin population analyses. An inspection of DOS curves reveals that the states near the Fermi level, *E*_F_, originate to a large extent from the Cu-3*d* atomic orbitals with minor contributions from the Te-5*p* atomic orbitals. These orbitals contribute to the heteroatomic Cu−Te and homoatomic Cu−Cu interactions within the layers of the [Cu@Te_4_] and [Cu@Te_6_] units. An examination of the −pCOHP curves brings to light that the largest percentage contributions to the net bonding capabilities arise from the Cu−Te interactions. The Cu−Cu interactions correspond to evidently smaller percentages to the net bonding capabilities than the Cu−Te interactions, because the heteroatomic interactions are related to a higher number (42) of bonds per unit cell and –IpCOHP/bond values (<−IpCOHP/bond> = 0.618 eV) than the homoatomic interactions (27 contacts/cell), whose –IpCOHP/bond values do not exceed 0.282 eV. Notably, the Cu−Cu and Cu−Te interactions change from bonding to antibonding states below the Fermi level—a feature [53,54] typically encountered for such interactions due to the nature of the overlap of the Te-5*p* and Cu-3*d* orbitals. Additionally, the Na−Te interactions (18 contacts/unit cell) show relatively small –IpCOHP/bond values (<−IpCOHP/bond> = 0.390 eV), which means that these states are less populated relative to the Cu−Te interactions due to the electron-donor character of sodium. Accordingly, the Na−Te interactions should be regarded as rather ionic bonds due to the evident electron transfer, while the Cu−Te separations should be viewed at as mixed-metal-like bonds (the term “mixed-metal-like” has been used as the Cu−Te interactions resemble characteristics of late-TM−post-TM bonds [32] in several polar intermetallics).

**Figure 3:**
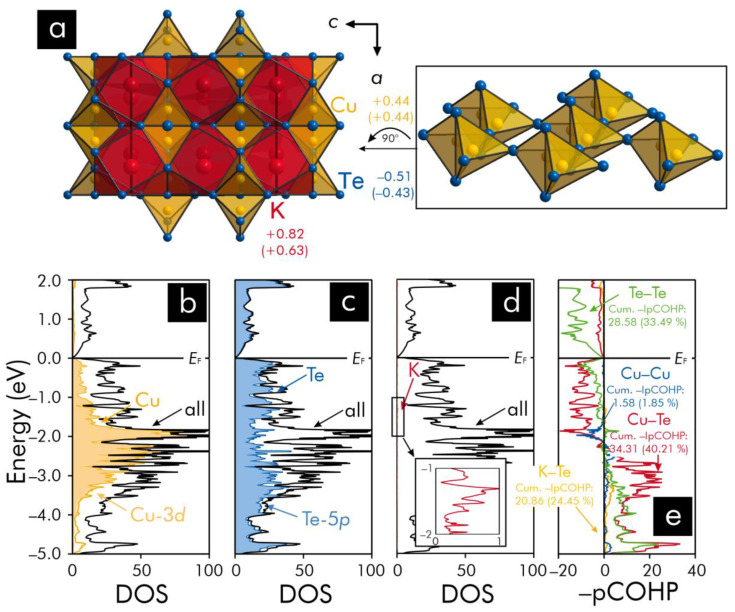
(**a**) Representation of the crystal structure of K_2_Cu_2_Te_5_: the tellurium polyhedra surrounding the copper and potassium atoms are shown in the insets, while the respective averaged Mulliken and Löwdin charges (in parentheses) have been included. (**b**–**d**) total, atom-, and orbital-projected densities-of-states (DOS) curves: the orbital-projected DOS are related to those states providing the largest contributions to the respective atom-projected DOS near the Fermi level, *E*_F_, that is represented by the black horizontal lines and (**e**) projected crystal orbital Hamilton populations (pCOHP): the respective cumulative –IpCOHP/cell values and their percentages to the net bonding capabilities have been included.

In the crystal structure of K_2_Cu_2_Te_5_ (Figure 3), each copper atom resides in the center of a tellurium tetrahedron, which is connected via one common edge to a neighboring [Cu@Te_4_] tetrahedron within [Cu@Te_4_]_2_ dimers. In addition, the dimers share common tellurium vertices with the nearest neighboring [Cu@Te_4_]_2_ dimers such that as sheets of dimers, [Cu@Te4]∞2, are constructed parallel to the *ab* plane. In the dimers, there are short Cu−Cu contacts (*d* = 2.574(2) Å), which, based on the atomic covalent radius of Cu (see above), point to the presence of possible Cu−Cu interactions. Furthermore, each [Cu@Te4]∞2 layer encloses additional tellurium atoms, of which each tellurium atom is closely located (*d* (Te−Te) = 2.781(1) Å) to two tellurium atoms of the respective [Cu@Te4]∞2 layer. Under consideration of the Te−Te distances previously [55] determined for tellurium dumbbells, such short Te−Te contacts imply that the additional tellurium atoms within the layers could be part of tellurium trimers, ((Te_3_)^2−^). Notably, there are also short Te−Te separations (*d* (Te−Te) = 2.798(1) Å) between the apical tellurium atoms of neighboring [Cu@Te4]∞2 layers, which suggest that the layers are connected via tellurium dumbbells, ((Te_2_)^2−^). The potassium atoms are surrounded by bicapped square tellurium antiprisms located between the [Cu@Te4]∞2 layers that are stacked along the *c* axis.

In summary, an inspection of the crystal structure for K_2_Cu_2_Te_5_ reveals that each unit cell is supposed to comprise four tellurium dumbbells and four tellurium trimers, which coordinate to the potassium and copper atoms. Since each unit cell contains four formula units, an application of the Zintl−Klemm concept to K_2_Cu_2_Te_5_ would result in an electron-precise distribution of valence-electrons according to the formula (K^+^)_2_(Cu^+^)_2_((Te_2_)^2−^)((Te_3_)^2−^); however, an examination of the Mulliken and Löwdin charges (Figure 3) indicates that the charges computed for copper and tellurium differ evidently from the charges predicted by the Zintl−Klemm treatment. In this connection, it should also be noticed that the charges of the tellurium atoms that are located in the middle of each (Te_3_) trimer (Mulliken: −0.17; Löwdin: −0.10) are smaller than those of the remaining tellurium atoms (Mulliken: −0.59; Löwdin: −0.52)—a tendency that is also indicated by applying the Zintl−Klemm formalism. Yet, the outcome of the population analyses suggests an absence of full valence-electron transfers, which is further corroborated by an examination of the DOS and −pCOHP of K_2_Cu_2_Te_5_.

An examination of the DOS curves for K_2_Cu_2_Te_5_ (Figure 3) shows that the states close to the Fermi level largely stem from the Cu-3*d* and Te-5*p* atomic orbitals, which contribute to the heteroatomic Cu−Te as well as homoatomic Te−Te and Cu−Cu interactions. Among those interactions, the largest percentage contributions to the net bonding capabilities correspond to the Cu−Te and Te−Te interactions. Furthermore, the K−Te interactions are related to smaller percentages of the net bonding capabilities than the Cu−Te and Te−Te interactions, while the number of the K−Te contacts per unit cell (80) is significantly higher than those of the Cu−Te (32) and Te−Te (12) separations. This is because the latter interactions are related to higher –IpCOHP/bond (<−IpCOHP/bond_Cu−Te_> = 1.072 eV; <−IpCOHP/bond_Te−Te_> = 2.382 eV) values than the K−Te interactions (<−IpCOHP/bond> = 0.261 eV). Such small –IpCOHP/bond values mean that the K−Te states are less populated because of the electron-donor character of potassium and, hence, should be considered as rather ionic interactions. Since the Cu−Te and Te−Te states correspond to higher –IpCOHP/bond values relative to the K−Te separations and comprise the majority of the bonding populations, it may be concluded that these interactions should be regarded as mixed-metal-like (Cu−Te) and covalent (Te−Te) bonds. The Cu−Cu –IpCOHP values (<−IpCOHP/bond> = 0.395 eV; 4 contacts/unit cell) are indicative of a net bonding character; however, the Cu−Cu interactions (such as the Cu−Te interactions do) change from bonding to antibonding states below the Fermi level—a feature [53,54] that has also been previously encountered for such interactions. An additional inspection of the characteristics at the Fermi level in K_2_Cu_2_Te_5_ also reveals that the Fermi level falls in a pseudogap, which typically [56,57,58] points to an electronically favorable situation.

**Figure 4:**
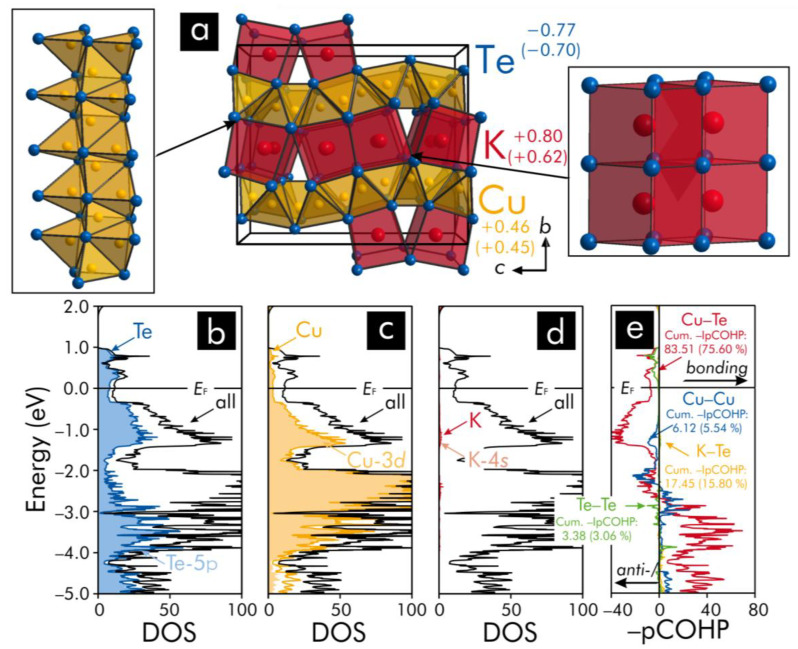
(**a**) Representation of the crystal structure of K_2_Cu_5_Te_5_: the tellurium polyhedra, which enclose the copper and potassium atoms and are condensed to the layers, are shown in the insets. Furthermore, the respective averaged Mulliken and Löwdin (in parentheses) charges have been included; (**b**–**d**) total, atom- and orbital-projected densities-of-states (DOS) curves: the orbital-projected DOS correspond to those states providing the largest contributions to the respective-atom-projected DOS near the Fermi level, *E*_F_, which is represented by the black horizontal lines and (**e**) projected crystal orbital Hamilton populations (pCOHP): the respective cumulative—IpCOHP/cell values and their percentage contributions to the net bonding capabilities have been included.

In the crystal structure of K_2_Cu_5_Te_5_ (Figure 4), the copper atoms reside in the centers of tellurium tetrahedra, which are linked via common vertices into [Cu@Te4]∞1 chains along the *a* and *c* axis. These [Cu@Te4]∞1 chains are condensed via common edges to puckered sheets that are stacked along the *b* axis. The potassium atoms are enclosed by tetragonal tellurium prisms, which share common faces within [K@Te8]∞1 chains and are packed between the puckered [Cu@Te4]∞2 sheets. Furthermore, each [K@Te8]∞1 chain is condensed via common faces to its nearest neighboring [K@Te8]∞1 chain thereby assembling [K@Te8]∞1 double chains that are connected via common edges to the two nearest [K@Te8]∞1 double chains. Notably, there are short Te−Te separations (*d* = 2.960(1) Å) between the [K@Te8]∞1 double chains that are tilted relative to each other such that the layers of the double chains are puckered. Under consideration of previous research [55] on tellurides containing tellurium dumbbells, such short Te−Te contacts typically point to the presence of tellurium dumbbells ((Te_2_)^2−^). Moreover, there are also short Cu−Cu contacts between the copper atoms (*d* = 2.687 (2) − 2.909(2) Å) within the puckered [Cu@Te4]∞2 sheets implying the presence of Cu−Cu closed-shell interactions.

Applying the Zintl−Klemm concept to K_2_Cu_5_Te_5_ suggests that a mixed-valence state could be evident for that telluride based on an electron-precise valence-electron distribution according to the formula (K^+^)_2_(Cu^+^)_4_(Cu^2+^)(Te^2−^)_3_((Te_2_)^2−^). In addition to the aforementioned formula, the formal valence-electron distributions could also be represented by the alternative formulae (K^+^)_2_(Cu^+^)_5_(Te^1.67−^)_3_((Te_2_)^2−^) or (K^+^)_2_(Cu^+^)_5_(Te^2−^)_3_((Te_2_)^2−^)(h^+^) (note that h^+^ represents an electron hole). Since the distributions of the valence-electrons cannot be reliably predicted based on the Zintl−Klemm treatments, using the Zintl−Klemm formalism to relate the valence-electron distributions to the structural features should be regarded with concern for this particular telluride. Accordingly, it can be inferred that the bonding nature in K_2_Cu_5_Te_5_ could be more complicated as suggested by the aforementioned Zintl−Klemm treatments—a circumstance that is also indicated by the Mulliken and Löwdin population analyses, because the charges computed for copper and tellurium (Figure 4) are much smaller than those charges predicted by the application of the Zintl−Klemm concept. Because the Mulliken and Löwdin charges are very similar for all copper atoms (Mulliken: +0.44 to +0.48; Löwdin: +0.45 to +0.46), it can be concluded that the Mulliken and Löwdin charges somewhat differ for the various tellurium atoms, if the charges are actually different for one of the constituting elements (notably, the Mulliken and Löwdin charges of all potassium atoms are the same). Indeed, the charges of the tellurium atoms of the Te_2_ dumbbells (Mulliken: −0.60; Löwdin: −0.54) are smaller than the charges of the remaining tellurium atoms (Mulliken: −0.89; Löwdin: −0.81).

Another hint that suggests that a Zintl−Klemm treatment could be problematic for this telluride is evident from an examination of the DOS curve for K_2_Cu_5_Te_5_ (Figure 4): although the Fermi levels in Zintl phases are typically located in band gaps [59], the characteristics at its Fermi level show that K_2_Cu_5_Te_5_ should be a metal being in agreement with previous [54] research on that telluride. The states around the Fermi level in K_2_Cu_5_Te_5_ largely stem from the Cu-3d atomic orbitals with minor contributions from the Te-5p atomic orbitals. Hence, it may be inferred that the majority of the bonding interactions arises from contributions of the aforementioned atomic orbitals—an outcome that is also indicated by an inspection of the –pCOHP curves and their respective integrated values (Figure 4).

An examination of the –pCOHP curves and the –IpCOHP values for K_2_Cu_5_Te_5_ shows that the largest percentages to the net bonding capabilities correspond to the Cu−Te interactions. Although the number of the K−Te contacts (64) is just slightly lower than that of the Cu−Te separations (80), yet, the K−Te interactions are related to a much lower percentage contribution to the net bonding capabilities than the Cu−Te interactions. This is because the Cu−Te –IpCOHP/bond values (<−IpCOHP/bond> = 1.044 eV) are much higher than those of the K−Te interactions (<−IpCOHP/bond> = 0.273 eV). Such small K−Te −IpCOHP values indicate that these states are less populated because of the electron-donor character of potassium—an outcome that has also been previously reported [60] for potassium-containing polar intermetallics. Since the Cu−Te −IpCOHP values are much larger than those of the K−Te interactions, the former interactions correspond to a more bonding character relative to the latter interactions. Hence, the K−Te interactions should be depicted as rather ionic interactions, while the Cu−Te interactions show characteristics of mixed-metal-like bonds.

The –IpCOHP values of the homoatomic Te−Te (<−IpCOHP/bond> = 0.844 eV) and Cu−Cu (<−IpCOHP/bond> = 0.153 eV) are indicative of a net bonding character for these interactions; however, the former homoatomic contacts have rather low contributions to the net bonding capability because of the small number (4) of Te−Te contacts. Although the number of the Cu−Cu contacts (40) is much higher than that of the Te−Te separations, the former interactions also correspond to rather small percentages to the total bonding capabilities. This is because the Cu−Cu interactions change from bonding to antibonding states below the Fermi level—a characteristic [53] of closed-shell interactions resulting in the rather small –IpCOHP/bond values (note that the term “closed-shell interactions” can be used, because the Mulliken and Löwdin population analyses of K_2_Cu_5_Te_5_ indicate that the charges of the copper atoms are smaller than +2 and even +1). Additionally, the Cu−Te interactions traverse from bonding to antibonding states as encountered for the two other ternary tellurides inspected in the present contribution (see above).

## 4. Conclusions

Understanding the nature of bonding in solid-state materials is decisive, because the bond energy has significant contributions to the total electronic ground state energy, which influences how atoms order in solids, and the bonding characteristics at the Fermi level of a given material could influence its chemical and physical properties. In the cases of tellurides, which are of great relevance in diverse fields of basic research and technologies, the structural features have typically been related to the distributions of valence-electrons by applying the Zintl−Klemm formalism. Yet, under consideration of ongoing research efforts [27,28,29,30] on the bonding nature of tellurides containing transition-metals, should such tellurides be assigned to the family of polar intermetallics or to the group of Zintl-phases, which are traditionally [20] solely composed of main group elements? To answer this question, we followed up with explorations of the bonding nature for the ternary NaCu_3_Te_2_, K_2_Cu_2_Te_5_, and K_2_Cu_5_Te_5_, which were reported in the present contribution.

From the analyses of the Mulliken and Löwdin charges, it is clear that there are no full valence-electron transfers as predicted by the Zintl−Klemm treatments. Namely, the Mulliken and Löwdin charges computed for the copper and tellurium atoms by means of quantum-chemical techniques are evidently smaller than the charges predicted by the Zintl−Klemm treatments. An examination of the –pCOHP curves and their respective integrated values revealed that the majority of the bonding interactions resides between the Cu−Te contacts that should be depicted as mixed-metal-like bonds. The alkaline-metal−Te interactions are less populated relative to the Cu−Te interactions because of the electron-donor character of the alkaline-metal atoms such that these interactions should be depicted as rather ionic bonds. The Cu−Cu −IpCOHP values indicate that these contacts comprise weak, but evident bonding interactions—a circumstance that has also been previously recognized [61] for copper-containing chalcogenides.

In conclusion, should tellurides containing transition-metals be assigned to the family of polar intermetallics or the group of the Zintl phases? While the limits of the Hume-Rothery phases have been well established [62,63] based on the valence-electron concentrations and the respective quantum-chemical elaborations of intermetallics (Figure 1), identifying the frontier between polar intermetallics and Zintl phases has remained challenging. Under consideration of the valence-electron concentrations recently [32] proposed for polar intermetallics, one should expect NaCu_3_Te_2_ (*e*/*a* = 2.67), K_2_Cu_2_Te_5_ (*e*/*a* = 3.78), and K_2_Cu_5_Te_5_ (*e*/*a* = 3.08) to be polar intermetallics. This expectation is further corroborated by our bonding analyses: the valence-electrons have been (largely) transferred from the electropositive alkaline-metal atoms to the copper-tellurium networks, which comprise mixed-metal-like Cu-3*d*−Te-5*p* interactions and closed-shell Cu-3*d*−Cu-3*d* interactions—a bonding pattern that is well known from polar intermetallics.
